# Physiology

**Published:** 1859-11

**Authors:** S. Weir Mitchell

**Affiliations:** Lecturer on Physiology in the Phila. Med. Association


### PHYSIOLOGY.

BY S. WEIR MITCHELL, M.D.,

LECTURER ON PHYSIOLOGY IN THE PHILA. MED. ASSOCIATION.

I.—*On the Natural Constants of the Healthy Urine of Man.* By the REV. S. HAUGHTON, F.R.S., Fellow of Trinity College, Dublin.

The author of this paper starts with the idea of Bernouilli, that the total quantity of work of which a healthy man is capable is constant, no matter in what description of labor he may engage. After partially admitting the justice of this statement, he gives the following propositions as offering a more correct view of the subject:—

1. A man in health consumes, per day, a certain amount of food and drink, and excretes, per day, an equal amount of waste matter, supposing him to be full grown, in good health, and not gaining or losing flesh.

2. The excretions are more highly oxidated than the food or drink consumed.

3. The difference of oxidation of the *ingesta and excreta,* converted into work, should account for, and be equal to, the amount of total labor effected per diem.

4. The work done by the body per day is—first, that expended on the organic functions; second, in sustaining the animal heat; third, the mechanical work, or bodily labor; fourth, the unknown, and hitherto unmeasured, mental labor.

The author then classifies the excretions, and proceeds to examine that of the urine with reference to mechanical labor and mental activity. He thus examines:—

1. The daily healthy discharge of urea.

2. That of uric acid.

3. That of phosphoric acid.

4. He compares the foregoing with the daily work, mental and physical, and the amount of food taken daily by the persons examined.

As to urea, he obtained the following results in six well-fed, flesh-eating, wine-drinking men:—

| | Urea, per day. grains. | Urine, per day. ounces. | Sp. gr. | Urea, per fl. oz. grains. | Solids, per day. grains. |
|---|---|---|---|---|---|
| Mean...... | 575·87 | 47·3 | 1021·7 | 12·18 | 1013·49 |

This agrees with Lehman and Bischoff's estimate of their own excreta. The next table gives the results of a like examination of urea in vegetarians, all of whom, however, included eggs and milk, or both, in their vegetarian diet list:—

| | Urea, per day. grains. | Urine, per day. ounces. | Sp. gr. | Urea, per fl. oz. grains. | Solids, per day. grains. |
|---|---|---|---|---|---|
| Mean...... | 393·99 | 59 | 1014·71 | 6·76 | 890·99 |

Mr. Haughton found, in the next place, that beef eaters excreted 4·55 grains of uric acid daily, and vegetarians 1·48 grains. Phosphoric acid gave the following results: For beef eaters, 37·07 grains; for vegetarians, 26·70 grains daily.

The author concludes this portion of his paper with an argument against the view which he supposes to be generally held, that the waste of nervous matter is the exclusive source of phosphoric acid. In the concluding section of his paper Mr. Haughton will apply the facts here stated to the elucidation of the problems set forth in the beginning of this analysis of his direct results.—(*Dublin Quarterly Journ. of Med. Science,* No. lv. 1859.)

II.—*On some Actions performed by Voluntary Muscles, which, by habit, become Involuntary.* By J. OSBORNE, M.D., King's Professor of Materia Medica.

This paper contains some curious observations, which, though of no great value, admit of being applied to explain certain peculiarities of diseased conditions:—

*Habits during sleep.*—In four boarding-schools of boys and girls, mostly under fourteen years of age, the number of those who slept on the right side was 248; on the left side, 260; and on the back, 234. In two other establishments, containing young persons of both sexes, but above the age of childhood, 23 slept on the left side, 58 on the right side, and 29 on the back. In a barrack, inspection of the sleepers during three nights gave the following results: On the left, 22; on the right, 44; on the back, 62.

If these returns be correct, they show with increasing years an increasing tendency to sleep on the right side. On comparing this with Pelletan's report of the frequency of pneumonia on the right side, we find the cases of right pneumonia to left pneumonia as 2½ to 1; and in Andral's table the same, or nearly the same statement is found. This conformity between the position generally assumed in sleep and the prevalence of inflammation in the right lung, seems to the author to justify him in connecting them together, as, *to some extent*, cause and effect.

The author adds, that we lie on the right side to relieve the viscera of the weight of the liver and to allow the heart greater freedom. He also states, that this preference of the right side in sleeping is not confined to man.

The remainder of the essay treats of the bent position in sleep, of snoring, of waking habits, of crying, as a relief in suffering. Among other points, the author has gone so far as to estimate the number of winks performed during given periods, and states that the eyelids are closed, on an average, four hundred and twenty-four times in an hour; but, as we might suppose, far less frequently when the attention is fixed on some object of interest.—(*Dublin Quarterly Journ. of Med. Science*, No. lv. 1859.)

### III.—*Observations on the Action of Pancreatic Juice on Albumen.* By WM. BRINTON, M.D.

In the last number of this journal we gave in full Dr. Corvisart's defence of his views in regard to the action of the pancreatic juice and pancreatic infusions upon albumen. Dr. Brinton, who has lately examined so much of the subject as relates to the effect of artificial infusions of pancreas in dissolving albumen, has reached the conclusion that these infusions owe their solvent power to putrefaction, and that, when perfectly fresh and prepared rapidly in cool weather, they possess scarcely any influence of the kind alluded to. The solvent power of the infusion was always at a minimum when it was just made, and at a maximum when putrefaction was complete. Dr. Brinton found that the process occupied from two to six times as long as was the case when gastric juice was the solvent employed; and he is finally of opinion, that Dr. Corvisart's experiments in nowise prove that the pancreatic juice is endowed with the power to dissolve albuminous matters in the digestive canal. Since, however, as he himself admits, he has not followed Corvisart in the vivisections with which he has further sustained his views, he has scarcely acquired the right to speak so decidedly upon the main subject in question.—(*Ibid.*)

### IV.—VULPIAN *on the Effects of Direct Excitation of the Liver and Kidney.*

M. Vulpian experimented in the following manner: An animal was killed by curare, and the action of the heart sustained by artificial respiration. The abdomen being opened, and the liver exposed to view, it was mechanically irritated by passing the point of a pin rapidly along its surface, so as to excite, but not to wound; the slight furrow thus made disappeared at once, and a line of vascular injection took its place. At the same time a new furrow formed, and along its centre a narrow elevation of the tissues was observed, corresponding to the line traced by the pin-point. Similar effects were attained to in experiments upon rabbits, dogs, etc.

M. Vulpian also observed, that the mechanical stimuli were better for observations of this nature than any galvanic arrangement—a fact also noted by M. Brown-Séquard some time since. A like series of researches upon the capsule of the kidney gave similar, or but slightly different results. The results thus obtained M. Vulpian ascribes principally to the irritability of the small vessels beneath the covering of the glands in question, while he believes, also, that the experiments indicate the existence of contractility in the extra-vascular tissues of the several organs here spoken of.—(*Comptes Rendus de la So. de Biol.*, 1858, p. 8.)

V.—*On the Reactions of the Medullary Substance of the Supra-renal Capsules.* By M. VULPIAN.

M. Vulpian, following up the researches of Virchow, Harley, and M. Colin, on the effect of reagents on the supra-renal capsules, observes that an infusion of the medullary substance gives, with the salts of iron, a deep blue or violet tint; and that a large number of substances produce in it a rose color—among these are iodine, chlorine, bromine, alkalies, etc. M. Cloez, who assisted M. Vulpian, failed to isolate chemically the substance which produced these reactions.

Whatever may be this material it is found in all animals, and reveals itself by characteristic behavior with certain tests. It exists only in the medullary portion of the gland, and is a product of the secretion of the organ. Its presence enables us to distinguish these from all other organs, no one of which gives the same reactions.

The author thinks that it is quite certain that the supra-renal capsules are blood glands, and that they pour into the circulation a product of secretion peculiar to themselves. The supra-renal capsules do not become fully active until after birth.—(*Comptes Rendus de la So. de Biol.*, Paris, 1858, p. 14, *et seq.*)

VI.—*Notes on the Phenomena which occur in the Tails of the very young embryo of Frogs, when they have been detached from the body.* By M. VULPIAN.

Spallanzani has stated that when the tail of the tadpole is cut off a process of regeneration takes place, after which the tail is reproduced. This experiment has been often tested, and is constantly successful. I have seen the new part form again after it had been several times taken off. Until now, physiologists have rarely made it a matter of research as to what became of the tail after it was cut off. Moreover, no results can be obtained by operating on well-developed tadpoles. In this condition the tail loses all movement as soon as it is separated from the body, unless the section have been made close to the base, because it may then contain a very small portion of the spinal marrow, which in the tadpole passes a little beyond the posterior portion of the body; the tail, at all events, soon becomes decomposed. The case is not similar when the experiment is made upon the very young embryo of the frog, at the time when their gills begin to form. The tail detached from the body lives some time, and interesting phenomena occur in it. I discovered this when it was too late to procure any more frogs' eggs, so that I was unable to vary the experiment in many ways, which I intend to do next year. The tails detached from the bodies frequently live several days, manifesting their existence by the motions which they make when stimulated, or when they are exposed to contact with the air, which appears to be a source of irritation. These motions consist in a series of flexions and a lifting up of the tail as in the act of swimming. But life is also manifested by yet more important and singular phenomena. At the time when the experiment begins the tail is formed of the central axis and of two membranous portions, one inferior, the other superior, the whole constituting the caudal fin. All the elements in these different portions are in their first stage; the epithelial cells, the muscular fibres, the nervous fibres, the vessels, and the blood. Without entering into details, which I shall enlarge upon in another publication, I may say that these tissues, examined daily, gradually attain perfection; they lose their fœtal character, the elements are multiplied, and, at the same time, the vitelline granulations which they inclose disappear completely. A cicatrice is formed in the neighborhood of the section; a new portion is here added to the tail, and this portion, which may be about as one-eighth to the whole length of the tail, seems younger; it is more transparent, and has no central axis, and the cells are filled with a larger number of granulations. This growth of cells takes place at the expense of the pre-existing cells; there is no intussusception. The tail flattens out without becoming larger. The vessels are seen to ramify; star-shaped cells give birth to capillary blood-vessels and probably to lymphatics; cutaneous pigment cells appear. The groups of muscles which are separated by intersections, and which form a large portion of the axis, are more clearly defined; these intersections are crossed by distinct vessels which give rise to a net-work of membranous lamina; in these vessels are seen stationary blood-globules, which become modified while the tail lives. In the only experiment which I could extend to any length, a tail, cut upon the 9th of April, 1858, was yet alive on the 27th; that is to say, at the end of eighteen days; but it was then almost dead, and was sacrificed for more complete examination. At the time the tail was cut off the blood-globules were entirely round, almost colorless, and very granular. During the last days of its existence the granulations had diminished in number, and were exceedingly fine; several globules were oval, and at the last they became of a marked yellowish tint.

I wished to make these experiments upon the embryos of the triton, but I could not succeed. They offer less resistance, and the cohesion of their elements is not so strong, so that they fall rapidly into decay. Nevertheless, I preserved the tail of the larva of the triton, which lived six days; the section had separated from the body not only the tail, but also the posterior half of the larva. No real scar was produced, but drawings, made every day with the camera, showed considerable changes in the length and shape of the caudal portion of the segment. The elements of this portion underwent notable modifications; but the length of the experiment did not admit of their being as profound as those in the elements of the tail of the embryo of the frog.

Thus the tail of the embryo of frogs, detached from the body, may live twenty days, and be the seat of the most incontestable vital phenomena. This cicatrization which occurs, this new portion which is reproduced, are tendencies to restoration. As in animals still lower, life, at this stage, undergoes segmentation, if we may be allowed the use of such a phrase. But as the vital force begins to resume its functions, the elements which it renders active are multiplied and perfected, and gradually attain to a higher organization. Circulation thus becomes an imperious necessity, either to convey new materials or to carry away the tissues already broken down. Finally, a multitude of minute molecules are deposited in the midst of the tissues, and life becomes extinct.—(*Comptes Rendus de la Société de Biologie*, 1858, p. 31.)

